# Looking beyond the brain to improve the pathogenic understanding of Parkinson’s disease: implications of whole transcriptome profiling of Patients’ skin

**DOI:** 10.1186/s12883-016-0784-z

**Published:** 2017-01-10

**Authors:** Anu Planken, Lille Kurvits, Ene Reimann, Liis Kadastik-Eerme, Külli Kingo, Sulev Kõks, Pille Taba

**Affiliations:** 1 North-Estonian Medical Centre, Sütiste Rd.19, Tallinn, 13419 Estonia; 2 Faculty of Medicine, University of Tartu, Tartu, Estonia; 3 Institute of Pathophysiology, University of Tartu, Tartu, Estonia; 4 Department of Dermatology, University of Tartu, Tartu, Estonia; 5 Dermatology Clinic, Tartu University Hospital, Tartu, Estonia; 6 Department of Neurology and Neurosurgery, University of Tartu, Tartu, Estonia

**Keywords:** Parkinson’s Disease, Neurodegeneration, Gene expression, High-throughput RNA sequencing, Skin-brain crosstalk

## Abstract

**Background:**

Parkinson’s Disease is a progressive neurodegenerative disease, characterized by symptoms of motor impairment, resulting from the loss of dopaminergic neurons in the midbrain, however non-neuronal symptoms are also common. Although great advances have been made in the pathogenic understanding of Parkinson’s Disease in the nervous system, little is known about the molecular alterations occurring in other non-neuronal organ systems. In addition, a higher rate of melanoma and non-melanoma skin cancer has been observed in the Parkinson’s Disease population, indicating crosstalk between these diseases.

**Methods:**

To understand the molecular pathogenesis and gene expression alterations of Parkinson’s Disease in peripheral tissues, and in order to explore the possible link between skin cancer and neurodegeneration, whole transcriptomic profiling of patients’ skin was performed. Skin biopsies from 12 patients and matched controls were collected, and processed with high-throughput RNA-sequencing analysis.

**Results:**

This analysis resulted in a large collection of over 1000 differentially expressed genes, among which clear biological and functional networks could be distinguished. The central functional processes altered in patients skin can be grouped into six broad categories: impaired cellular metabolism and mitochondrial dysfunction, defective protein metabolism, disturbed skin homeostasis, dysfunctional nuclear processes, altered signalling and tumour pathways, as well as disordered immune regulation.

**Conclusions:**

These results demonstrate that the molecular alterations leading to neurodegeneration in the CNS are systemic and manifest also in peripheral tissues, thereby indicating the presence of “skin-brain” crosstalk in Parkinson’s Disease. In addition, the extensive homeostatic imbalance and basal stress can lead to increased susceptibility to external and internal mutagenic hazards in these patients, and thus provide a possible molecular link for the crosstalk between skin cancer and Parkinson’s Disease.

**Electronic supplementary material:**

The online version of this article (doi:10.1186/s12883-016-0784-z) contains supplementary material, which is available to authorized users.

## Background

Parkinson’s disease (PD) is a progressive neurodegenerative disorder characterized by symptoms of resting tremor, rigidity, bradykinesia and postural instability. The motor symptoms of PD are considered to result from the loss of substantia nigra dopaminergic neurons, however patients experience also numerous non-motor symptoms, due to involvement of central and peripheral organ systems [1]. The non-motor symptoms are frequently under-recognized and under-treated, although they greatly impact the life quality of PD patients and can often be the first indication of PD pathology, proceeding the onset of motor-symptoms by several years [2], thus it has been suggested that PD should no longer be viewed solely as a dopamine-mediated basal ganglia disease, but rather as a multisystem progressive disorder [3]. The pathogenesis of PD is complex and likely caused by a deregulated network of interconnected pathways, including mitochondrial dysfunction, bioenergy failure, oxidative stress, defective protein processing, endoplasmatic reticulum stress, as well as decreased levels of neurotrophic growth factors and activation of immune mechanisms [4, 5]. The traditional hallmark of PD is the presence of α-synuclein aggregates (Lewy Bodies-LB) in the CNS, however these aggregates have been shown to manifest also in the peripheral nervous system. Numerous studies have investigated the pathogenic processes occurring in the nervous system, however the reflection of disease pathology in the peripheral tissues, has been poorly characterized. In addition, due to lack of diagnostic and prognostic biomarkers a definitive diagnosis for PD can only be performed post-mortem, thus information on PD pathogenesis, with possible outlook on novel biomarkers or disease-modifying therapy, is of great value.

PD patients present with several skin symptoms including cutaneous neuropathy, seborrhoea, hyperhidrosis and impaired wound healing [6]. A further link to skin involvement in PD has been provided by studies demonstrating the presence of cutaneous denervation and α-synuclein deposits in dermal somatosensory and autonomic nerve fibres [7, 8]. The extent of skin denervation and aggregate deposition, is considered to correlate with LB pathology in the brain [7], rendering easily obtainable skin biopsy samples useful for pathogenic studies and for novel biomarker discovery. The hypothetical existence of a link between cancer and neurodegeneration has been under debate for some years, and is based mostly on epidemiological observations of patients with neurodegenerative diseases, who in general exhibit a reduced incidence of many common types of cancers. This holds true for PD, where a negative interaction between most cancers and PD has been established, however an increased risk for both melanoma and non-melanoma skin cancer has been found to be strongly associated to PD, as epidemiological studies have demonstrated an up to 4 fold increased risk for melanoma and non-melanoma skin cancers [9–11] in these patients. This crosstalk seems to be bidirectional as the diagnosis of cutaneous melanoma is also associated with a 2 fold increased risk for subsequent development of PD [12]. The causal relationship and underlying molecular mechanisms between these two diseases have remained unclear, however suggested common mechanisms include dysfunction in melanin/neuromelanin pathways, tyrosine metabolism, α-synuclein pathology, activation and deregulation of the cell cycle, but also the genetic aspect as familial PD gene variations have been identified in skin cancers [13–15].

There are indications that PD also manifests in peripheral tissues, as demonstrated by skin symptoms, dermal denervation and α-synuclein deposition, however the precise molecular basis for PD pathology in skin is unknown. In addition, the increased risk of skin cancer in PD patients suggests that common deregulated pathways exist between these diseases, however the exact pathogenic mechanisms for this crosstalk have remained unclear. Numerous gene expression profiling studies have been performed on different PD models, including animal models and human post-mortem brain tissues, however none of the studies have evaluated the gene expression patterns in peripheral solid tissues of PD patients. In our study we utilized large-scale transcriptomic analysis from whole skin puncture biopsy material, which represents the gene expression signal from all cells of epidermal and dermal layers. The aim of the current study was to test the hypothesis that the generalized biomolecular defect observed in the nervous system is systemic and present also in non-neuronal peripheral tissues, and to analyse whether these alterations could provide evidence for the crosstalk between PD and skin cancer.

## Methods

### Study subjects and ethics

The study was conducted in accordance with the Declaration of Helsinki and approval was granted by the Tartu University Ethics Committee. Informed consent was obtained from all patients and controls. 12 PD patients who fulfilled the Queen Square Brain Bank diagnostic criteria [16, 17] and 12 healthy controls were selected for RNA sequencing. The demographic data, history of disease and clinical data were documented and a summary of participant characteristics is presented in Additional file 1: Table S1. Disease severity, disability and cognitive state were assessed using validated instruments including the Movement Disorders Unified Parkinson’s Disease Rating Scale (MDS-UPDRS) [18, 19], the Hoehn and Yahr Scale (HY) [20], the Schwab and England Activities of Daily Living Scale (SE-ADL) [21] and the Mini Mental State Examination (MMSE) [22]. The presence of familial PD and cancers were excluded for all patients at the time of inclusion. In addition, all concomitant medications were documented and none of the patients were taking any medications other than the commonly used dopaminergic medications. The RNAseq control group consisted of 6 males (mean age 70 years) and 6 females (mean age 73 years). To assess the medical status and to exclude any history of neurodegenerative disease, a medical interview was performed for all control patients. Validation analysis of RNA sequencing data was performed on 37 patients of which 18 were males (mean age 69.3 years) and 19 females (mean age 70 years) and 32 controls of which 15 were males (mean age 68.5 years) and 17 females (mean age 68.2 years). The clinical characteristics of the PD patient cohort used for qRT-PCR analysis is presented in Additional file 1: Table S9.

### Experimental design, tissue sampling and RNA extraction

One 4 mm punch-biopsy specimen was taken from non-sun-exposed skin of each subject from both study groups. All biopsy specimens were instantly frozen in liquid nitrogen and stored at -80C° until RNA extraction. Biopsies were homogenized with Precellys24 homogenizer with the Cryolys system (Bertin Technologies). RNeasy Fibrous Tissue Mini Kit (Qiagen) was used for total RNA extraction, according to the manufacturer’s protocol. During the purification on-column DNase I treatment was performed (Qiagen). The RNA quality was assessed using Agilent 2100 Bioanalyzer, the RNA 6000 Nano kit (Agilent Technologies) and the Qubit fluorometer (Life Technologies). The lowest RIN of study samples was 6.7.

### Whole transcriptome sequencing (RNA-seq)

Fifty nanogram of each total RNA sample was amplified with Ovation RNA-Seq System V2 Kit (NuGen) and the output double stranded DNA was used to prepare SOLiD 5500 System DNA fragment libraries according to manufacturers’ protocols (LifeTechnologies). For library preparation the barcoding adapters were used and 12 libraries were pooled prior sequencing. For sequencing the SOLiD 5500 XL platform and paired-end sequencing chemistry was applied (75 bp in forward and 35 bp in reverse directions). In case of 12 samples per three flowchip lanes approximately 40 million mappable reads were expected per one sample, which is enough for successful whole transcriptome expression pattern analysis.

### Real-Time quantitative PCR

For validation of RNA sequencing data total RNA from 37 patients and 32 controls (including the 12 + 12 samples from RNAseq) was converted to cDNA using random primers and High Capacity cDNA Reverse Transcription Kit with RNase Inhibitor (Applied Biosystems). Duplex quantitative real-time PCR (qRT-PCR) analysis was performed using TaqMan Gene Expression Assays with VIC (housekeeping gene ActinB) and FAM (gene of interest) probes and TaqMan® Gene Expression Master Mix (Applied Biosystems). The TaqMan® Gene Assay IDs were the following: Hs01060665_g1 (*ActinB*), Hs00761940_s1 (*SAA-1*), Hs00754237_s1 (*SAA-2*), Hs00361191_g1 (*HBA-2*), Hs00758338_g1 (*CALML-6*), Hs00819920_mH (*DGCR-6 L*), Hs01012810_g1 (*CST E/M*), Hs00962118_g1 (*OR2HR*), Hs00603977_m1 (*ROMO-1*), Hs00936068_m1 (*ADAMDEC*), Hs01891339_s1 (*HCRT*), Hs01652462_m1 (*KLRC-3*), Hs00155790_m1 (*APOC-1*). RT-PCR was performed using ABI PRISM 7900HT Fast Real-Time PCR System equipment (Applied Biosystems) and the ABI PRISM 7900 SDS 2.2.2 Software. Each reaction was made in four replicates to minimize technical errors.

### Data analysis and statistics

For analysis of RNAseq data Lifescope software was used. For statistical analysis DeSeq package for R [23] was used to test for differential expression by use the negative binomial distribution and a shrinkage estimator for the distribution’s variance. Package performs samples comparison and also adjusts P-value to overcome multiple testing problem. DeSeq package uses Benjamini-Hochberg procedure, which controls for false discovery rate (FDR). Functional pathway analysis was performed using the QIAGEN’s Ingenuity® Pathway Analysis (QIAGEN Redwood Citytool), followed by manual classification of the selected genes into broad functional categories. Based on the 10 major functional networks provided by Ingenuity Pathway Analysis we narrowed our framework of manual classification into 6 broad functional categories in association to Parkinson’s Disease, for which we used Pubmed searches initially for the role of the specific gene and then searching for the association with keywords such as “Parkinson’s Disease” “neurodegeneration” “neuro” “Alzheimer’s Disease”, “brain”. Each gene was categorized only once, according to the more prominent functional role in association to Parkinson’s Disease.

For analysis of real-time PCR the samples were normalized to the corresponding housekeeping gene Actin-B and the comparative ΔΔCT method was used to calculate the fold change for all the samples.

## Results

### Differential expression of genes in PD skin

**Fig. 1:**
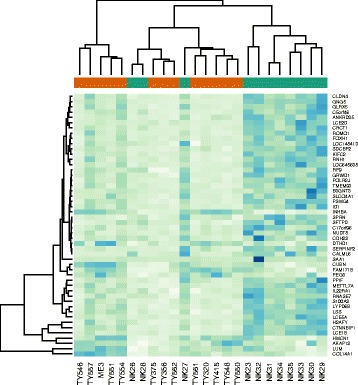
Hierarchical clustering (heat-map) demonstrating the expression profiles of the top 50 differentially regulated genes in Parkinson’s disease (TY-, ME- *red*) versus normal skin (NK - *green*). Gene expression levels are presented as colour variations from *dark blue* (high expression) to *pale green* (low expression) for each individual sample (columns) and for each gene (rows)

The RNA sequencing analysis of PD and control skin resulted in 1074 genes to be differentially regulated between control and PD samples, with a FDR ≤ 0.05. A heatmap of the 50 most significantly changed genes in the PD versus control sample is shown in Fig. 1. Most of the altered genes in our study showed decreased expression in the PD samples: 82% (877 genes) down- and 18% (197 genes) up-regulated. The Ingenuity Pathway Analysis resulted in 10 major functional networks to be influenced by PD including (Table 1.): 1) gene expression, protein synthesis, dermatological disease and conditions (reference score of 46, with 28 focus molecules differentially expressed between PD and normal skin); 2) dermatological diseases and conditions, immunological disease and inflammatory disease (35/23); 3) cellular assembly and organization, behaviour, cell signalling (34/23); 4) cancer, immunological disease, cellular development (33/22); 5) connective tissue disorder, dermatological diseases and conditions, developmental disorder (30/21); 6) lipid metabolism, molecular transport, small molecule biochemistry (28/21); 7) molecular transport, neurological disease, psychological disorders (28/20); 8) cellular movement, haematological system development and function, immune cell trafficking (21/18); 9) cellular growth and proliferation, haematological system development and function, tissue development (21/16); 10) lipid metabolism, small molecule biochemistry, vitamin and mineral metabolism (19/16). Due to the limitations of the computer-based functional analysis, which managed to classify only a small proportion of genes under specific categories, we performed further manual classification of the differentially expressed genes, which resulted in 6 broad functional categories including: cellular metabolism/mitochondrial dysfunction (23% of genes); protein metabolism/transport (16%); skin homeostasis (11%); regulation of nuclear processes (12%); cellular signalling and tumorigenesis (7%); immunological processes (7%) and others. A full list of gene expression data for all genes in our study can be found in Additional file 2.

**Table 1:** Top 10 functional network functions affected in Parkinson’s disease versus normal skin as analysed by Ingenuity Pathway Analysis. The first number in the score column reflects the number of genes in a reference gene set and the second reflects the number of focus genes altered in our study

	Associated Network Function	Score/ nr of genes
1	Gene expression, Protein synthesis, Dermatological diseases and conditions	46/28
2	Dermatological diseases and conditions, Immunological disease, Inflammatory disease	35/23
3	Cellular assembly and organization, Behaviour, Cell signalling	34/23
4	Cancer, Immunological disease, Cellular development	33/22
5	Connective tissue disorder, Dermatological diseases and conditions, Developmental disorder	30/21
6	Lipid Metabolism, Molecular Transport, Small Molecule Biochemistry	28/21
7	Molecular Transport, Neurological Disease, Psychological Disorders	28/20
8	Cellular Movement, Haematological System Development and Function, Immune Cell Trafficking	21/18
9	Cellular Growth and Proliferation, Haematological System Development and Function, Tissue Development	21/16
10	Lipid Metabolism, Small Molecule Biochemistry, Vitamin and Mineral Metabolism	19/16

### Impaired cellular metabolism and mitochondrial dysfunction

The largest group of genes altered in PD skin involves the regulation of cellular metabolism and mitochondrial dysfunction, with 252 genes classified in this category, most of them being downregulated in PD (see Additional file 1: Table S2). Apparent from these results is the severe defect in mitochondrial respiration, as 36 genes of the mitochondrial respiratory complex (out of 96 genes) were supressed, including complex I (18 out of 44), complex III (5 out of 10), complex IV (7 out of 19) and complex V (6 out of 19). Of special note in regard to the metabolic alterations in PD skin, is the increased expression of peroxisome proliferator-activated receptor gamma coactivator-1α (*PPARGC1A or PGC-1α*), which has been considered to be the central inducer of mitochondrial biogenesis in mammalian cells. In addition, many genes in the cellular metabolism category are associated with oxidative/peroxidative metabolism and the antioxidant response, with 25 of the down-regulated genes playing a role in this process, highlighted by deregulation of central oxidative stress genes such as glutaredoxin 2 and 5, cystatin E/M and B, glutathione peroxidase and transferases, different peroxiredoxins and reactive oxygen species modulators. In addition several metallothioneins were downregulated in PD skin. Only two of oxidative stress response mediators were up-regulated: glutathione S-transferase-mu1 and peroxidasin homolog. Another large group of genes differentially expressed in PD skin is associated with fatty acid metabolism, with 30 of the down- and only 3 of the up-regulated genes falling into this metabolic pathway. Processes involved include all aspects of fatty acid metabolism including synthesis and degradation by beta-oxidation, as well as binding and transport. Also 3 members of the phospholipase A2 group were down-regulated in PD samples, indicating dysfunctional phospholipid digestion and metabolism in PD. Other affected cellular metabolic processes include the oxidation of aldehydes, central mitochondrial transport genes, differential expression of mitochondrial ribosomal proteins (12 members), purine/pyrimidine metabolism, steroidogenesis, glucose/carbohydrate, amino acid and iron/metal metabolism and detoxification.

### Impaired protein metabolism

Impaired protein aggregation and degradation in the nervous system has been linked to PD by many studies. We hereby demonstrate widespread defects in protein metabolism occurring in PD patient’s skin, with 170 genes being affected (see Additional file 1: Table S3.). Similarly to the above mentioned metabolic processes, most of the genes in this group also showed decreased expression in PD (155 vs 15). Among these were genes regulating protein translation, including 8 eukaryotic elongation and initiation factors, a large group of ribosomal proteins (26 genes) and several genes mediating post-translational modification. Of special note, in relation to PD pathogenesis, is the deregulation of a group of genes which play a role in protein folding and the unfolded protein response, as well as ER and Golgi protein trafficking and transport proteins. Also several of the downregulated genes play a role in vesicular transport. In addition, a group of axonemal motor proteins showed differential expression with light chain dyneins and myosin being downregulated and heavy chain dyneins being upregulated in PD. In addition, protein degradation by the ubiquitin-proteasome system (UPS) is impacted in PD skin, including neddylation and ubiquitilation of proteins, as well as proteasomal degradation, as demonstrated by downregulation of 11 members of the proteasome complex. Only one member of the ubiquitilation process showed increased expression. Normally the impaired protein aggregation and degradation initiates the autophagic-lysosomal response in cells, however in PD skin several central markers of autophagy were also supressed. In addition, several phosphatases, proteases and peptidases, as well as peptidase inhibitors were decreased. Of note among the dysregulated peptidases are members of the ADAM metallopeptidase family, members of the matrix metallopeptidase family and calpain peptidases. Also several members of the serine/serpine peptidase inhibitors were downregulated in PD skin. In addition to the above mentioned proteases also cathepsins and cysteine protease inhibitors were downregulated in PD skin.

### Impaired skin homeostasis in PD patients

Our study identified a group of 115 genes regulating epidermal and dermal homeostasis to be affected in PD patients (see Additional file 1: Table S4.). In accordance with the overall suppression of gene expression, most of these genes were downregulated in PD. In regard to epidermal renewal, a large group of genes participating in keratinocyte cytodifferentiation was altered in PD skin, including downregulation of 9 different keratins and several keratinocyte differentiation factors. Ephrins also influence the process of keratinocyte proliferation and differentiation and in our study Ephrin-A1 showed downregulation, whereas EphA6-receptor showed upregulation in PD skin. Another large set of genes affected in PD skins involved the process of epidermal cornification and desquamation, as demonstrated by the suppression of 20 genes from the epidermal differentiation complex (EDC) including loricrin (the most abundant gene in the cornified envelope), members of the small proline rich proteins, the S100 protein family and late cornified envelope proteins. The three members of the stratified epithelium secreted peptides complex (SSC)—dermokine, suprabasin and keratinocyte differentiation-associated protein, were also downregulated in PD skin. Another downregulated pathway related to cornification in our study, includes cystatins, cathepsins as well as transglutaminase. Adding to the complexity of epidermal homeostasis and permeability are intercellular junctions which regulate the flow of molecules and pathogens in epithelium. We also observed altered regulation of several claudins and defensins, which are central players in the structure and maintenance of tight junctions, and which also play a role in the antimicrobial properties of the epidermis and form a part of the CE. In addition dermokine—a protein known to be part of a the stratified epithelium secreted peptide complex, functioning in keratinocyte differentiation and possibly playing a role in inflammatory response—was downregulated in PD samples. A few melanocytic genes were also downregulated, including d-dopachrome tautomerase and macrophage migration inhibitory factor, which both play a role in eumelanin biosynthesis. In regard to the dermal layer of skin, multiple members of the collagen family showed increased expression in PD skin, whereas a few collagens showed decreased expression. In addition, a large group of genes playing variable roles in cytoskeletal dynamics and morphology, including the regulation of actin and tubulin morphology were altered. Lastly, the antimicrobial defence mechanisms seem also to be affected in PD skin, as demonstrated by down-regulation of several defensins, mucins and rnases.

### Nuclear processes influenced in PD skin

A large set of differentially regulated genes (128 genes) in PD skin play a role in nuclear processes and epigenetic regulation (see Additional file 1: Table S5.) 14 genes participating in different aspects of the cell cycle were supressed in PD, including cyclins, cyclin dependent kinases (CDKs) and other cell division cycle associated genes. Also 14 regulators of basal transcription of RNA were downregulated, with different RNA polymerase II polypeptide associated factors, activators of basal transcription and elongation factors being supressed. Another important category supressed in PD is related to the reparation and degradation of nuclear and mitochondrial DNA, with 11 genes including endo- and exo-nucleases, dnases and growth-arrest associated molecules down-regulated. A large group of transcription factors were deregulated in PD skin, several of these controlling various aspects of skin homeostasis, proliferation and differentiation, but others regulating energy metabolism, cellular signalling and immune responses. In addition, a large set of altered genes can be associated to epigenetic regulation. This is highlighted by the down-regulation of achaete-scute homolog 2, which is known to regulate various aspects of epigenetics. Also genes related to chromatin remodelling and DNA binding, transcriptional/post-transcriptional modification and RNA splicing were differentially regulated, as well as a large group of miRNAs, snRNPRs and snoRNAs.

### Signalling pathways and tumour genes influenced in PD skin

We observed a basal deregulation of cellular signalling pathways and suppression of several oncogenes and tumorsupressors in PD skin (see Additional file 1: Table S6.). Several of the central growth and survival pathways were also altered in PD skin, as highlighted by suppression of *Ras* signalling by decreased expression of central *Ras*-effectors. Also *small-GTPase* family genes and *G-protein* signalling were affected. In addition, central effectors of *Wnt* signalling and several regulators of *NOTCH* signalling showed altered levels in PD skin. PD also impacts fibroblast growth factor, insulin-like growth factor, transforming growth factor-β, nuclear factor-κβ, and other central signalling proteins, such as carcinoembryonic antigen proteins, epidermal growth factor and vascular endothelial growth factor family proteins.

### Immune pathways affected in PD skin

The most statistically significantly changed gene in our study was serum amyloid A1 (*SAA1*), which together with *SAA2* (also downregulated), are known to be major acute phase proteins, participating in inflammation, but also in various other processes such as cholesterol metabolism and amyloid aggregation. Also a large set of chemokines, cytokines and cluster of differentiation molecules were altered in PD skin (see Additional file 1: Table S6.). Also the tumor-necrosis-factor (*TNF*) family signalling was induced in PD skin, with several *TNF* family members differentially regulated. Further in regard to humoral immunity, genes related to the HLA complex showed differential regulation, such as the *HLA-DQA2* which was significantly upregulated in PD skin, of special interest being that changes in the HLA genes have been associated with an increased risk for sporadic PD. Also several complement genes, immunoglobulins, interleukins and interferon signalling molecules were altered in PD skin. Lastly the components of cellular immunity including T-cell signalling, showed alterations in PD skin.

### Validation of a gene set by qRT-PCR

A subset of 12 genes showing differential expression in PD skin samples was selected for validation of RNA sequencing data by qRT-PCR, in a larger set of patient and control samples. These genes were chosen due to the highest differential expression levels in PD samples, but also because of their potential interest in pathogenic pathways in PD. The RNAseq and qRT-PCR levels for the following genes are shown in Additional file 1: Table S8: *SAA-1*,*-2*, hemoglobin α-2 (*HBA-2*), calmodulin-like 6 (*CALML-6*), DiGeorge syndrome critical region gene 6-like (*DGCR-6 L*), cystatin E/M (*CST E/M*), olfactory receptor family 2 subfamily H member 2 (OR2HR), reactive oxygen species modulator 1 (*ROMO-1*), ADAM-like decysin 1 (*ADAMDEC*), hypocretin (orexin) neuropeptide precursor (*HCRT*), killer cell lectin-like receptor subfamily C member 3 (*KLRC-3*), apolipoprotein C-1 (*APOC-1*).) Although the exact gene expression levels varied between the two methods, 9 out of 12 genes were changed in the similar direction.

## Discussion

The transcriptomic analyses of PD patients skin revealed a large set of over 1000 differentially regulated genes. Interdependent biological and functional networks can be distinguished among the differentially regulated genes and the deregulation of these networks demonstrates a state of severe impairment in basal homeostasis, as well as decreased defence mechanisms to cellular stress in PD skin. Our study results correlate with, and further enhance, the existing knowledge of PD associated pathogenesis and additionally provide a possible molecular explanation for the association of PD with melanoma/non-melanoma skin cancers.

### “Skin-Brain” crosstalk in PD – disturbed mitochondrial and protein homeostasis in skin

A focal point of our study highlights the relevance of “skin-brain” crosstalk in understanding the pathogenic mechanisms of neurodegenerative diseases, such as PD. Our findings support the assumption that the biomolecular alterations of PD are systemic and are also reflected in other non-neuronal cells of the body, demonstrating the applicability of skin biopsies as an easily accessible ex vivo solid-tissue based model system, for downstream pathogenic studies and for possible diagnostic/biomarker discovery. Notably, our study highlights that PD reflection in skin is dominated by global suppression of central cellular processes (over 80% of genes were downregulated), indicating a state of extensive cellular stress and compensatory molecular changes limiting the physiological functioning to minimum.

The current understanding of PD pathogenesis in the nervous system, is led by impairment of two major biological systems: mitochondrial dysfunction and protein metabolism. One of the most robust findings in our study, is the evident basal defect in cellular bioenergetics, metabolism and the impairment of mitochondrial function. This is initially demonstrated by the suppression of 1/3 of the components of the mitochondrial electron transfer chain in PD skin, including complexes I, III, IV and V. In addition, the cellular redox homeostasis, oxidative stress and antioxidant response are supressed in PD skin. Mitochondrial impairment in PD skin is also demonstrated by downregulation of genes responsible for mitochondrial dynamics and transport. Adding to the impairment, is the suppression of fatty acid biosynthesis and metabolism. PD has been associated with lipid disturbances, including the specific vulnerability of fatty acids to oxidation, leading to an increase in ROS production and enhanced susceptibility to oxidative stress, but also in relation to the direct interactions of α-synuclein with lipids, as its aggregation is known to occur in the presence of lipid membranes and free fatty acids [24]. Other impaired central biosynthetic and metabolic processes include oxidation of aldehydes, purine and pyrimidine metabolism, steroidogenesis, amino acid, glucose and carbohydrate metabolism, as well as calcium, iron and glycoprotein metabolism. It is noteworthy, that although most of the effector molecules in the cellular metabolism pathways were downregulated in our study, we observed up-regulation of the transcriptional coactivator and central inducer of mitochondrial biogenesis—*PGC-1α*, which controls oxidative metabolism through expression of genes in the mitochondrial respiratory chain, and regulates detoxification of reactive oxygen species [25]. Our finding is in line with a large genome-wide meta-analysis on PD brain samples, which found *PGC-1α* to be the main common pathway to be deregulated between 17 different microarray sets, with most of the *PGC-1α* controlled genes being suppressed in PD samples [26]. In addition, our study observed regulation of other transcription factors related to bioenergetics, indicating that the compensatory changes for disturbed bioenergetics and cellular metabolism in PD skin are regulated on the level of central transcriptional control. In addition, mitochondrial DNA regulation has been associated with PD and decreased expression of mitochondrial polymerase –*POLRMT*, an enzyme which plays a fundamental role in expression and replication of the human mitochondrial genome and in mitochondrial protein translation, was observed in our study. We also noted decreased expression of several mitochondrial ribosomal proteins, which function as components of the mitochondrial ribosome and regulate the translation of all the essential polypeptides of the oxidative phosphorylation system. Based on the current study results, it can be concluded that a “basal” transcriptomic defect in cellular bioenergetics and metabolism exists in PD skin, this finding correlating with the known understanding of the pathogenic processes of PD in CNS.

The second most robust finding in our study, is the involvement of genes related to the processes of protein metabolism, transport and degradation. Imbalances of protein homeostasis have been associated with PD pathogenesis in the nervous system, however our study demonstrates that protein homeostasis in PD skin is affected already at the level of ribosomal biogenesis and basal eukaryotic translation, as seen by downregulation of a large set of different ribosomal proteins, as well as several eukaryotic translation initiation and elongation factors. The downregulation of ribosomal proteins in response to stress, can be considered compensatory for limitation of energy expenditure and in order to maintain essential cellular functioning, however long-term inhibition can lead to severe cellular damage and death [27]. Further, a large group of genes playing a role in protein post-translational modifications, protein folding, aggregation and processing in the ER, are affected in PD skin. Another large group includes proteins participating in the cellular trafficking of proteins, vesicles and organelles. Further, the protein degradation machinery of the UPS is evident in PD skin, highlighted by suppression of several proteins involved in ubiquitination and neddylation, as well as downregulation of 11 subunit components of the proteasome, proteasome assembly chaperones and maturation proteins. In addition, several central genes of the autophagic-lysosome cascade are affected, demonstrating impaired autophagic response in PD skin. Also, a large group of cellular and lysosomal phosphatases, peptidases and proteinases are co-ordinately deregulated in PD skin, indicating defective protein degradation machinery and impaired proteolysis. Taken together our findings demonstrate a severe “basal” impairment of protein homeostasis in PD skin, characterized by suppression of protein translation, cellular trafficking, as well as dysfunctional protein quality control by the UPS and impairment of the autophagic response, which all contribute to the vicious cycle of misfolded protein buildup, further contributing to cytotoxicity.

### Impaired skin homeostasis, nuclear processes and tumorigenic pathways—the mechanistic link for predisposition to skin cancer in PD patients?

The second focal point of our study enhances the understanding of the mechanistic association between skin cancer and PD, as demonstrated by a basal defect in skin homeostasis, deregulated nuclear processes, as well as dysbalanced cellular signalling, tumorigenic pathways and inflammatory processes—all these alterations possibly contributing to the specific vulnerability of PD skin to mutagenic hazards (such as UV radiation, somatic mutations, genomic instability), which can provide the basis for the mechanistic link to the increased risk of skin cancer in this patient population.

Our study data demonstrates direct alteration of skin physiology in PD patients, characterized by dysregulation of epidermal renewal/keratinocyte differentiation, cornification/desquamation, response to injury and stress, as well as altered structural and molecular composition of dermis. We observed differential regulation of a large group of keratins and several keratin-related proteins, which indicates impaired epithelial differentiation, tissue fragility and structural integrity of PD skin. Our study revealed a coordinated suppression of parallel pathways of the cornification and desquamation processes, highlighted by the suppression of the EDC, which contains 57 genes crucial for the differentiation process located within a tight cluster on chromosome 1q21 (20 genes of EDC supressed) and also ephrin A1, which is a central regulator of epidermal growth, located in close proximity to the EDC on chromosome 1q arm. In addition, we observed the decreased expression of all genes of the stratified epithelium-secreted peptide complex, as well as the cystatin/cathepsin/transglutaminase pathway, which regulates the cross-linking of the CE proteins and influences the desquamation of the stratum corneum. Further we observed suppression of several different junction and desmosome proteins and deregulation of the antimicrobial defence in PD, indicating that the desmosomal adhesions and anchoring junctions are defective in PD, thereby contributing to impairment of structural integrity and barrier function. In addition, the dermal components of skin were affected, characterized by altered levels of several members of the collagen family, as well as deregulation of cytoskeletal remodelling and dynamics, these changes contributing to impairment of tissue elasticity, predisposing to premature aging of skin, and impacting the structural and compositional remodelling. In conclusion, maintenance of skin homeostasis, thru a balanced orchestration of regeneration, cell renewal, differentiation and senescence, is essential to withstand stress and mutagenic hazard and disruption of this equilibrium can lead to skin disease, such as development of cancers.

The second significant category of differentially expressed genes, with possible impact in both PD and skin cancer involves nuclear regulation of cellular processes in PD skin. This is highlighted by suppression of cell cycle proteins, such as several cyclins, *CDKs*, activators/inhibitors of *CDKs* and others. In skin, cell cycle regulation is essential to maintain the homeostatic balance between proliferation and differentiation, whereby keratinocytes respond to cell cycle insults and DNA damage by deregulation of the cell cycle and induction of terminal differentiation [28], however chronic dysregulation can lead to enhanced predisposition for development of cancers. Further, our study indicates that several regulators of basal transcription, and a large group of central transcription factors are deregulated in PD skin, which act as direct regulators/corepressors of genes regulating epidermal terminal differentiation (including the EDC), epithelial-mesenchymal transition, programmed cell death, oxidative stress and tumorigenesis. Other deregulated transcription factors play more variable roles regulating cell growth, proliferation, differentiation, longevity, and acting as downstream targets of multiple signalling pathways. Another important aspect of PD and skin cancer crosstalk can be drawn from the observed downregulation of genes related to DNA/mtDNA repair and degradation, which can contribute to buildup of damaged DNA, interfere with normal cellular functioning and also predispose to tumorigenesis. And lastly, in regard to nuclear regulation of cellular processes, a large group of genes in our study was also associated with the epigenetic regulation of gene expression, as seen by deregulation of proteins participating in chromatin remodelling, DNA binding, RNA/DNA processing and transcriptional/post-transcriptional modification, RNA splicing, as well as a set of different micro-RNAs, small- nuclear- and small-nucleolar-RNAs. The deregulation of cell cycle proteins and transcription factors, as well as the suppression of DNA repair processes and modification of epigenetic signalling, might be executed as a compensatory cytoprotective effect against various pro-apoptotic stimuli from the aspect of PD, however the chronic form of such stress can also provide the basis for molecular predisposition to different forms of skin cancers in these patients.

The third important category in our study, in relation to PD and skin cancer crosstalk, is associated to cellular signalling and tumorigenesis. We observed down-regulation of a large set of tumour suppressors and oncogenes in PD skin. Further many central intra- and inter-cellular signalling genes and growth factors were altered in PD skin, including *Ras* and other *small-GTPases*, *G-protein* signalling pathways, *WNT*, *NOTCH* and several others. Perhaps the most prominently affected signalling pathway in PD skin is related to *WNT* signalling, where deregulation of central *WNT* effectors, provides one possible mechanistic association between the processes of epidermal renewal/differentiation, tumorigenesis and neurodegeneration. *WNT* signalling plays a dominant role in controlling the patterning of skin, influencing the decision of stem cell lineage and in controlling the functioning of differentiated skin cells, and disruption of this signalling pathway has been associated with the development and progression of both melanoma and non-melanoma cancers [29]. On the other hand *WNT* signalling has been strongly associated with PD pathogenesis, functioning in midbrain dopaminergic neuron development, synaptic plasticity and transmission. In addition, suppression of several members of the Ras superfamily indicates dysfunctional growth, differentiation and survival mechanisms in PD skin, including cellular proliferation, cytoskeletal dynamics/morphology, membrane trafficking, cellular adhesion and vesicular transport.

And lastly, our study indicates dysregulation of immune pathways in PD skin. The interplay of pro- and anti-inflammatory signalling, and the discrimination of causal and effector changes in the context of both PD and cancer is complex, however chronic inflammation has been shown to be one of the main factors fostering all stages of neoplasia, but also one of the pathogenic processes in PD progression, thus the basal inflammatory dysfunction associated with PD can thereby contribute to increasing the risk of cancer development in these patients.

In regard to the existing explanations for the basis of PD and melanoma crosstalk, most studies have emphasized the role of the common skin- and neuro-melanin pathways during early development, and possible defects in tyrosine metabolism. Our study did not observe specific changes in regard to melanin pathways or tyrosine metabolism, however as our study design was set up to evaluate the gene expression changes occurring in whole skin of PD, and not specifically the small population of melanocytes, it can be that these changes remained too subtle for detection with our methodology. Follow-up studies utilizing gene expression profiling of cell-type specific samples, could provide further assistance in dissecting the PD related pathways in skin. In addition, it has been suggested that perturbations on the genetic level could contribute to the underlying crosstalk between melanoma and PD, although the majority of our study subjects (11/12 tested) were not carriers of mutations in any common PD associated genes (data not shown), the role of PD-associated genetic variants which can mediate the expression of quantitative trait locus effects in both skin and brain, cannot be excluded. One limitation of our study is the relatively low log2FC levels observed for gene expression, which might pose difficulties in distinguishing the true signal from noise, thus it cannot be excluded that some of the genes with milder expression levels in our pathway analysis might be attributable to noise. This however is a common finding in multiple ex vivo gene expression studies of a chronic disease and as all affected pathways in our study consisted of multiple genes with intertwining functions, the general conclusions can be considered to be valid.

## Conclusions

**Fig. 2:**
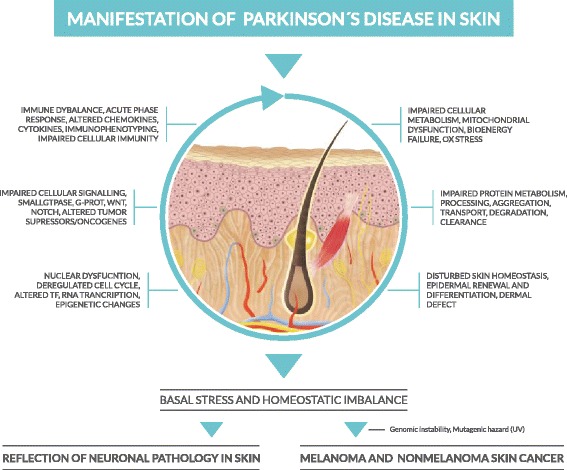
A schematic presentation of the manifestation of Parkinson’s Disease in skin, as characterized by an interplay of alterations affecting six central molecular processes, leading to basal cellular stress and homeostatic imbalance. These processes can be considered as the reflection of Parkinson’s Disease pathology in skin, but in the setting of internal and external mutagenic hazards, these alterations can also form the underlying basis for increased risk of skin cancers in these patients

In conclusion, our study (as shown in schematic Fig. 2.) demonstrates a large basal defect in cellular bioenergetics and mitochondrial dysfunction, impaired protein metabolism, dysbalanced skin homeostasis, deregulation of nuclear processes, as well as disturbances in many central signalling and immune pathways, indicates that the pathogenic processes associated with neurodegeneration are occurring also in peripheral tissues, such as skin. Further our study concludes that this severe “basal defect” in cellular homeostasis associated with the pathogenesis of Parkinson’s disease, could provide a mechanistic molecular basis for enhanced sensitivity of PD skin to patients internal (such as genomic instability) and external mutagenic hazards (such as UV radiation), thus leading to enhanced predisposition of PD patients for development of skin cancers and thereby providing a link for the crosstalk between neurodegenerative disease and cancer.

## Additional files

Additional file 1: Table S1. Demographic and clinical characteristic of patients with Parkinson’s Disease. * MMSE was not obtained from patient nr.5; PIGD, postural instability gait disorder; MDS-UPDRS, Movement Disorders Society Unified Parkinson’s Disease Rating Scale, scores range from 0 to 260 (higher scores indicating more severe disability; [18, 19]). Hoehn and Yahr stages from 1 to 5 (higher scores indicating more severe disability, [20]). Schwab and England scores range from 0 to 100 (lower scores indicating more severe disability; [21]). MMSE, mini mental state examination, scores range from 0 to 30 (scores under 24 indicating dementia; [22]). **Table S2.** The cellular and mitochondrial metabolism pathways affected in Parkinson’s disease versus normal skin. **Table S3.** The protein metabolism pathways affected in Parkinson’s disease versus normal skin. **Table S4.** The skin homeostasis pathways affected in Parkinson’s disease versus normal skin. **Table S5.** The nuclear pathways affected in Parkinson’s disease versus normal skin. **Table S6.** The signalling/tumorigenicity pathways affected in Parkinson’s disease versus normal skin. **Table S7.** The immune pathways affected in Parkinson’s disease versus normal skin. **Table S8.** Differentially expressed genes in Parkinson’s disease versus normal skin validated by qRT-PCR. Gene names: serum amyloid A-1 and A-2 (*SAA-1,-2*), haemoglobin α-2 (*HBA-2*), calmodulin-like 6 (*CALML-6*), DiGeorge syndrome critical region gene 6-like (*DGCR-6 L*), cystatin E/M (*CST E/M*), olfactory receptor family 2 subfamily H member 2 (*OR2HR*), reactive oxygen species modulator 1 (*ROMO-1*), ADAM-like decysin 1 (*ADAMDEC*), hypocretin (orexin) neuropeptide precursor (*HCRT*), killer cell lectin-like receptor subfamily C member 3 (*KLRC-3*), apolipoprotein C-1 (*APOC-1*).). **Table S9.** Demographic and clinical characteristic of patients with Parkinson’s Disease used in the qRT-PCR analysis. (DOCX 97 kb)
Additional file 2: The comparative gene expression list from RNA-sequencing analysis of skin samples from Parkinson’s Disease patients and controls. An excel file of all regulated genes between Parkinson’s Disease patients and control skin samples from RNA-sequencing analysis, which includes a column form of data including the geneID, log2FC, logCPM, p-value, FDR, the HGNC identifier and the name of the gene. (XLSX 1752 kb)

## Abbreviations

ADAMDEC: ADAM-like decysin 1
APOC-1: Apolipoprotein C-1
CALML-6: Calmodulin-like 6
CDKs: Cyclin dependent kinases
CE: Cornified envelope
CNS: Central nervous system
CST E/M: Cystatin E/M
DGCR-6L: DiGeorge syndrome critical region gene 6-like
EDC: Epidermal differentiation complex
ER: Endoplasmatic reticulum
FDR: False discovery rate
HBA-2: Hemoglobin α-2
HCRT: Hypocretin (orexin) neuropeptide precursor
HY: Hoehn and Yahr Scale
KLRC-3: Killer cell lectin-like receptor subfamily C member 3
LB: Lewy Bodies
MDS-UPDRS: Movement Disorders Unified Parkinson’s Disease Rating Scale
miRNAs: micro-RNAs
MMSE: Mini Mental State Examination
OR2HR: Olfactory receptor family 2 subfamily H member 2
PD: Parkinson’s disease
POLRMT: Mitochondrial polymerase
PPARGC1A/PGC-1α: Peroxisome proliferator-activated receptor gamma coactivator-1α
qRT-PCR: Quantitative Real-Time PCR
RIN: RNA integrity number
RNA-seq: RNA sequencing
ROMO-1: Reactive oxygen species modulator 1
SAA1/SAA2: Serum amyloid A1/A2
SE-ADL: Schwab and England Activities of Daily Living Scale
snoRNAs: Small nucleolar RNAs
snRNPRs: Small nuclear ribonucleo proteins
SSC: Stratified epithelium secreted peptides complex
TNF: Tumor-necrosis-factor
UPS: Ubiquitin-proteasome system
UV: Ultraviolet
