# Supplementary material for: Looking beyond the brain to improve the pathogenic understanding of Parkinson’s disease: implications of whole transcriptome profiling of Patients’ skin
Source: BMC Neurol. 2017 Jan 10;17:6. doi: 10.1186/s12883-016-0784-z (PMC5223462; doi:10.1186/s12883-016-0784-z)
Supplement: Additional file 1: Table S1. — Demographic and clinical characteristic of patients with Parkinson’s Disease. * MMSE was not obtained from patient nr.5; PIGD, postural instability gait disorder; MDS-UPDRS, Movement Disorders Society Unified Parkinson’s Disease Rating Scale, scores range from 0 to 260 (higher scores indicating more severe disability; [18, 19]). Hoehn and Yahr stages from 1 to 5 (higher scores indicating more severe disability, [20]). Schwab and England scores range from 0 to 100 (lower scores indicating more severe disability; [21]). MMSE, mini mental state examination, scores range from 0 to 30 (scores under 24 indicating dementia; [22]). Table S2. The cellular and mitochondrial metabolism pathways affected in Parkinson’s disease versus normal skin. Table S3. The protein metabolism pathways affected in Parkinson’s disease versus normal skin. Table S4. The skin homeostasis pathways affected in Parkinson’s disease versus normal skin. Table S5. The nuclear pathways affected in Parkinson’s disease versus normal skin. Table S6. The signalling/tumorigenicity pathways affected in Parkinson’s disease versus normal skin. Table S7. The immune pathways affected in Parkinson’s disease versus normal skin. Table S8. Differentially expressed genes in Parkinson’s disease versus normal skin validated by qRT-PCR. Gene names: serum amyloid A-1 and A-2 (SAA-1,-2), haemoglobin α-2 (HBA-2), calmodulin-like 6 (CALML-6), DiGeorge syndrome critical region gene 6-like (DGCR-6 L), cystatin E/M (CST E/M), olfactory receptor family 2 subfamily H member 2 (OR2HR), reactive oxygen species modulator 1 (ROMO-1), ADAM-like decysin 1 (ADAMDEC), hypocretin (orexin) neuropeptide precursor (HCRT), killer cell lectin-like receptor subfamily C member 3 (KLRC-3), apolipoprotein C-1 (APOC-1).). Table S9. Demographic and clinical characteristic of patients with Parkinson’s Disease used in the qRT-PCR analysis. (DOCX 97 kb) [file 12883_2016_784_MOESM1_ESM.docx]

*Supplementary Table 1.* Demographic and clinical characteristic of patients with Parkinson’s Disease.

| **Subject nr.** | **Sex** | **Age (years)** | **Age of onset**  **(years)** | **Disease**  **duration (years)** | **Subtype** | **MDS/**  **UPDRS** | **Hoehn and Yahr** | **Schwab and England** | **MMSE** |
| --- | --- | --- | --- | --- | --- | --- | --- | --- | --- |
| 1 | F | 83 | 74 | 9 | PIGD | 79 | 4 | 60 | 26 |
| 2 | F | 77 | 68 | 9 | Hypokinetic-rigid | 68 | 3 | 70 | 30 |
| 3 | F | 76 | 67 | 4 | Tremor | 77 | 3 | 80 | 27 |
| 4 | F | 68 | 65 | 4 | Tremor | 26 | 1.5 | 100 | 30 |
| 5* | F | 65 | 55 | 10 | PIGD | 114 | 4 | 60 | - |
| 6 | F | 69 | 67 | 3 | Tremor | 72 | 2.5 | 80 | 29 |
| 7 | F | 80 | 76 | 5 | Hypokinetic-rigid | 28 | 2 | 95 | 25 |
| 8 | M | 60 | 46 | 15 | Hypokinetic-rigid | 83 | 2.5 | 80 | 28 |
| 9 | M | 64 | 58 | 4 | Tremor | 40 | 2.5 | 90 | 30 |
| 10 | M | 67 | 64 | 4 | Hypokinetic-rigid | 65 | 2 | 80 | 29 |
| 11 | M | 81 | 80 | 2 | Tremor | 43 | 2.5 | 90 | 30 |
| 12 | M | 75 | 64 | 13 | PIGD | 105 | 4 | 70 | 30 |
| **Average** | **7F/5M** | **72.1** | **65.3** | **6.8** | **NA** | **66.7** | **2.8** | **79.6** | **28.5** |

*Supplementary Table 2.* The cellular and mitochondrial metabolism pathways affected in Parkinson’s disease versus normal skin. The gene names in bold text reflect the upregulated genes, while all others were downregulated.

| **MITOCHONDRIAL BIOGENESIS** | ATF3, CEBPA, CEBPB, **CREB5**, **PPARGC1A/PGCα1**, POLRMT |
| --- | --- |
| **MITOCHONDRIAL RIBOSOMAL PROTEINS** | MRPL14, MRPL15, MRPL23, MRPL27, MRPL33,, MRPL41, MRPL52, MRPL54, MRPL55, MRPS12, MRPS17,MRPS24 |
| **MITOCHONDRIAL ELECTRON TRANSPORT CHAIN** | COMPLEX I**:** NDUFA1, NDUFA2, NDUFA4, NDUFA7, NDUFA8, NDUFA11, NDUFA13, NDUFAB1, NDUFB1, NDUFB2, NDUFB10, NDUFB11, NDUFC1, NDUFC2, NDUFS5, NDUFS6, NDUFS7, NDUFV1  COMPLEX III**:** CYC1, UQCR10, UQCR11, UQCRC1, UQCRQ  COMPLEX IV**:** COX5B, COX6A1, COX6C, COX7A1, COX7A2, COX7A2L, COX7B  COMPLEX V**:** ATP5E, ATP5G2, ATP5G3, ATP5I, ATP5J2, ATP5L |
| **CELLULAR RESPIRATION/ENERGY METABOLISM** | **ADCY8, ADCYAP1**, ADCK2, ADCK3, ATPIF1, HINT2, **OXGR1,** SURF1 |
| **OXIDATIVE STRESS/ PEROXISOME/ PEROXIDATION/ ANTIOXIDANTS** | ATOX1, BLVRB, CCDC56, COQ4, CST6, CSTB, FDXR, GLRX2, GLRX5, GPX1, **GSTM1**, GSTO1, GSTO2, GSTP1, HAO2, HSPB2, MT1G, MT1L, MT1M, MT1X, MT2A, MT4, PAOX, PEX16, PRDX1, PRDX2, PRDX5, **PXDNL**, ROMO1, SEPW1, SNN, SPR, TST, TXNL4A, TXNRD2, VNN2 |
| **MITOCHONDRIAL DYNAMICS AND TRANSPORT MEMBRANE/ PORE/ FISSION** | ABCC8, FIS1, MTCH1, NKAIN2, PAM16, PPIF, SLC14A1, SLC25A20, SLC25A29, SLC25A5, SLC26A7, SLC34A2, SLC52A1, SLC9A4, SMCR7, TIMM10, TIMM13, TIMM8B, TOMM7 |
| **FATTY ACID BIOSYNTHESIS** | ACAA1, ACADVL, ACOX3, ACSBG1, ACSS1, **ADIPOQ**, APOC1, **ATP8A2**, AWAT2, CBR3, CES4A, COASY, CYB561D2, CYB5A, CYB5R1, DEGS2, DGAT2, ECHDC3, ELOVL1, ELOVL3, FA2H, FABP4, FABP7, **GLYATL2**, LBP, LEP, MLXIPL, MOGAT2, PLA2G2A, PLA2G2F, PLA2G3, PLIN2, PLIN3, PLIN4, PLIN5, PXMP2, SPHK1, **SPHKAP**, SPNS2, SREBF1, **TECRL** |
| **GLYCOPROTEIN METABOLISM** | **B3GALT5**, B3GNT3, **B3GNT7**, CLEC4E, FSTL3, **GALNT5**, GLTPD1, GP1BB, GPC1, GPC2, **GPC3**, **GPC6,** HS3ST4, HS3ST6, LFNG, LGALS1, LGALS12, LGALS7, LGALS7B, MGAT1, NAGLU, OSGEP, PSG9, **RELN**, RFNG, RHBG, SDCBP2, SGSH |
| **PURINE/PYRIMIDINE METABOLISM** | APRT, CKB, GUCY1B2, GUCY2D, GUK1, NME1, NME1-NME2, NME2, NME3, NT5C, NT5DC2, NUBP2, UPB1 |
| **AMINO ACID METABOLISM** | BCAT2, **CDO1**, HMGCL, HGD, FAH, GAMT, GGT5, GPT2, MPST, OAZ1, PRODH, **PRRG3**, THNSL2 |
| **OXIDATION OF ALDEHYDES** | ALDH16A1, ALDH1L1, ALDH3B1 |
| **STEROIDOGENESIS/ STEROID METABOLISM** | AKR1C3, CYP4F2, CYP4F8, HSD17B2, HSD17B8, LSS, MVD, MVK, PMVK |
| **GLUCOSE/ CARBOHYDRATE METABOLISM** | CSN1S1, FBP1, PMM1, TALDO1, TSTA3 |
| **CALCIUM HOMEOSTASIS** | CACNA1H, CALML3, CALML5, CALML6, **CAPS2**, CIB1, MS4A4A, ORAI1, S100A12, S100A16, S100A2, S100A4, S100A6, **S100A7A,** S100A9, SOLH/CAPN15 |
| **IRON/METAL METABOLISM** | SLC11A1, TF |
| **OTHER** | **ST6GALNAC5, ST8SIA6**, SULT1A1, **SULT1C4**, SULT4A1 |

*Supplementary Table 3.* The protein metabolism pathways affected in Parkinson’s disease versus normal skin. The gene names in bold text reflect the upregulated genes, while all others were downregulated.

| **EUCARYOTIC TRANSLATION** | EEF1A1P9, EEF1D, EIF1AY, EIF1B, EIF4A3, EIF4EBP1, ELL, FAU |
| --- | --- |
| **RIBSOSOMAL PROTEINS** | RPL11, RPL13, RPL21, RPL21P28, RPL22, RPL22L1, RPL23A, RPL27, RPL27A, RPL29, RPL35, RPL36A, RPL37A, RPL39, RPL39L, RPL41, RPL8, RPLP0, RPLP1, RPS12, RPS19BP1, RPS20, RPS29, RPS4Y1, RPS9, RRP9 |
| **POST-TRANSLATIONAL MODIFICATION** | ADPRHL2, GALNS, NUDT14, NUDT16L1, NUDT8, NAA10, NAT6 |
| **PROTEIN FOLDING/ ER PROTEINS/UPR** | CNIH4, DPM3, EBP, EBPL, ERP27, ERP29, OSGEP, PFDN2, PFDN5, SDF2, SDF2L1, SERF2, SEC61B, SEC61G, TRAPPC2L, TRAPPC4, TRAPPC6A, TMEM147, MANF |
| **VESICULAR TRANSPORT/ PROTEIN TRAFFICKING/ GOLGI PROTEINS/ ENDOSOMES** | ALS2CL, AP2S1, AP4M1, ARF5, CCDC64B, CLTB, CUTA, CYTH4, DNAH5, DNAH6, DNAJB2, DNAJC5, DNAL4, DYNLL1, EXOSC7, GALNTL2, GOLGA7B, KIFC2, LFNG, MGAT1, MYL6, RAB20, RAB40C, RIMS1, SCAMP3, **SCG2**, SLC35A2, SLC35D2, SNF8, SNX15, SPRN, SSR4, SYNGR2, **SYT13**, SYT8, TMED3, UNC119, VAMP5, VAMP8, **VAT1L**, VPS28, YIF1B |
| **UBIQUITILATION/ NEDDYLATION** | NEDD8, NOSIP, OTUB1, RBX1, REEP4, RNF126, RNF181, UBA52, **UBASH3A**, UBB, UBL4A, UBL5, UBXN1, USP6 |
| **PROTEIN DEGRADATION/ PROTEOSOME** | POMP, PSMA6, PSMB1, PSMB3, PSMB6, PSMB9, PSMC5, PSMD9, PSMG3, PSMG4, SHFM1/DSS1 |
| **AUTOPHAGY** | ATG16L2, ATG4D, **CA8**, MAP1LC3A, RAB24, ROBLD3, TSPO, ULK3 |
| **PHOSPHATASES** | ACYP1, DUSP2, DUSP22, DUSP23, ITPA, MDP1, PPP1R12C, PPP1R14B, PPP1R16A |
| **PROTEASES/ PEPTIDASES** | **ADAMDEC1, ADAMTS3**, ADAMTS15, ASPRV1, **CAPN6**, CAPN10, CAPN13, CLPP, ELANE, HTRA3, KLK11, KLK8, MMP3, **MMP16**, **PI16**, PLAU, PRSS3, SERPINA1, SERPINF2, SGSH, SPINK1, SPINT1, SPINT2, **TMPRSS11A**, **TMPRSS4**  TPSG1 |

*Supplementary Table 4.* The skin homeostasis pathways affected in Parkinson’s disease versus normal skin. The gene names in bold text reflect the upregulated genes, while all others were downregulated.

| **EPIDERMAL HOMEOSTASIS** | EFNA1, EPHA6, KRT15, KRT17, KRT27, KRT31, KRT4, KRT71, KRT74, KRT79, KRT85, **KRT9, KRTAP1-5, KRTAP4-1**, KRTAP5-10, KRTCAP2, KRTCAP3, **KC**, **LUM, UPK1A** |
| --- | --- |
| **EPIDERMAL DIFFERENTIATION COMPLEX** | LOR, **SPRR2A**, SPRR2B, SPRR2E, SPRR2G, **S100A7A**, S100A12, S100A16, S100A2, S100A4, S100A6, S100A9, LCE1A, LCE1B, LCE1C, LCE1D, LCE1E, LCE1F, LCE2C, LCE2D, LCE3D, LCE5A |
| **CORNIFICATION AND DESQUAMATION PATHWAY** | CST6, CSTB, CTSD, CTSF, CTSL1, TGM5 |
| **STRATIFIED EPITHELIUM SECRETED PEPTIDES COMPLEX** | DMKN, SBSN, KRTDAP |
| **DESMOSOMAL AND TIGHT JUNCTION PROTEINS** | CLDN4, CLDN5, CDH22, CTNNBIP1, **MPP4**, PKP3 |
| **DERMAL HOMEOSTASIS** | COL18A1, COL5A3, COL9A3**, COL14A1, COL1A1, COL1A2, COL24A1, COL3A1, COL6A5, COL6A6**, PSORS1C2 |
| **CYTOSKELETAL DYNAMICS AND MORPHOLOGY** | **ACTC1**, ARPC1B, ARPC5L, CAPG, COMP, CORO6, **FILIP1, KLHL4, LMOD3**, MGP, **MXRA5, MYOT, PCDH9**, TCAP, TMSB15B, TMSB4X, TNNT1, **TPPP**, TSPAN4, TTLL12, TUBA1C, TUBB2A, TWF2, UNKL |
| **ANTIMICROBIAL DEFENCE** | DEFA6, DEFB1, DCD, MUC1, **MUC16**, PNLDC1, RNASE2, RNASE7, RNH1, SFTPD |
| **MELANOCYTE SPECIFIC GENES** | DDT, MIF |

*Supplementary Table 5.* The nuclear pathways affected in Parkinson’s disease versus normal skin. The gene names in bold text reflect the upregulated genes, while all others were downregulated.

| **REGULATION OF CELL CYCLE** | CCND3, CCNF, CCNO, CDC34, CDCA3, CDK10, CDK5RAP3, CDKN1C, CHTF18, CKS2, G0S2, **HORMAD1**, MZT2B, ORC6, SERTAD1 |
| --- | --- |
| **REGULATION OF BASAL RNA TRANSCRIPTION** | ABT1, CPSF3L, DAZAP1, POLD4, POLR2G, POLR2H, POLR2J, POLR2L, POP5, POP7, SSU72, TAF1C, TCEB2 |
| **TRANSCRIPTION FACTORS/ IMMEDIATE EARLY RESPONSE** | ARGLU1, c-FOS, CITED4, CTBP1, DNASE1L2, E4F1, **ETV1**, ETV7, **FOXF1**, FOXF2, FOXH1, HOXB2, ID1, IER2, IER3, IER5, KLF2, OLIG3, **SIX4**, **TCF24**, TEF |
| **ZINC FINGER PROTEINS** | **ZC3H12D**, **ZNF114**, ZNF219, **ZNF319**, **ZNF334**, ZNF446, ZNF524, ZNF598, **ZNF713**, ZNHIT1, ZNHIT3 |
| **NUCLEAR RECEPTORS** | NR1H3, **NUP210L**, **RORB** |
| **CHROMATIN REMODELLING/DNA BINDING** | AES, CTBP1, **HFM1**, **HIST1H1D**, **HIST1H3C**, HMGN3, INO80C, PNLDC1, SSBP4 |
| **DNA REPAIR MECHANISMS/mtDNA REPAIR/ENDONUCLEASES** | AEN, DDIT4/REDD1, DNASE1L2, ENDOG, ENDOU, FANCE, GADD45B, GADD45GIP1, NEIL2, PNKP, TREX1 |
| **RNA DNA PROCESSING AND MODIFICATION/EPIGENETIC REGULATION** | ASCL2, COMTD1, CRNDE, DMAP1, DUS3L, LSM4, MED10, METTL7A, **PEG10**, **PEG3**, **PEG3-AS1**, PUF60, PUSL1, RNPS1, RP9, RUVBL2, SAP25, SF3B5, **SOX2OT**, TARBP2, TAX1BP3/TIP1, THOC6, TRPT1, **TTTY10**, WBSCR22 |
| **miRNAS/snRNPRs/snoRNAs** | MIR1244, MIR1282, MIR147B, MIR205, NHP2, NHP2L1, NOP10, SNRNP25, SNRNP70, SNRPB, SNRPD2, SNRPD3, SNRPE, SNRPF, SNHG5, SNHG9, SNHG8 |
| **OTHER** | CECR5, MUSTN1, QTRT1 |

*Supplementary Table 6.* The signalling/tumorigenicity pathways affected in Parkinson’s disease versus normal skin. The gene names in bold text reflect the upregulated genes, while all others were downregulated.

| **TUMOR SUPRESSORS/ ONCOGENES** | AIP, ASPSCR1, BLCAP, FAU/FBR-MuSV, FGR, GLTSCR2, LZTS2, MYEOV2,  OVCA2, PIM3, SPDEF, TP53TG1, TUSC1, YPEL3 |
| --- | --- |
| **REGULATION OF SIGNAL TRANSDUCTION** | **AKAP12**, DOK1, DOK2, **GRAP2**, LIN7B, PDZK1IP1, SIRPB1, **SPHKAP**, **STAP1**, STAP2, TNK2 |
| **RAS PATHWAY** | HRAS, PITX1, RAB20, RAB40C, RAB24, RASSF1, RASSF7, RIN1 |
| **SMALL GTPASE SIGNALLING** | AGAP3, ARAP1, ARHGAP40, ARHGEF10L, **ARHGEF38,** ARHGEF5, RAPGEF3, RHOB, RHOC, RHOT2, RHOV |
| **G-PROTEIN SIGNALLING** | GNG11, GNG5, **GPRC5A**, GPSM3, RAMP2, RGS10, SGSM3 |
| **WNT PATHWAY** | **APCDD1L**, CTNNBIP1, DKK1, DVL1, HESX1, WISP2, WNT11 |
| **NOTCH PATHWAY** | DLK1, HES5, RFNG |
| **FGF SIGNALLING** | CNPY1, **FGF7**, **FGF9**, FGFBP2, FGFR3 |
| **IGF SIGNALLING** | **IGF2**, IGFBP6, IGFL2, **INS-IGF2**, TMEM219 |
| **TGFβ SIGNALLING** | **ACVR1C, INHBA** |
| **NFκβ SIGNALLING** | **NKAPL**, TMEM101, CCDC22 |
| **OTHERS** | BOP1, BRMS1, CEACAM3, **CEACAM6**, CSNK2B, EGFL6, **GNRHR**, **HCCAT5**, **NHEG1**, **OGN**, **PTTG3P**, SCGB2A2, VEGFB |

*Supplementary Table 7.* The immune pathways affected in Parkinson’s disease versus normal skin. The gene names in bold text reflect the upregulated genes, while all others were downregulated.

| **ACUTE PHASE PROTEINS** | SAA1, SAA2 |
| --- | --- |
| **CHEMOKINES/ CYTOKINES/ INTERLEUKINS** | CCL15, CCL23, CCL27, **CCL5**, **CXCL13**, **CXCL17**, **CXCL9**, CKLF, CRLF1, IL1F10, **IL31RA**, TREM2 |
| **CD MOLECULES** | CD151, CD163, CD180, CD33, CD40, CD9 |
| **TNF SIGNALLING** | **EDA2R**, TMEM120A, **TNFSF14**, **TNFSF4**, TNFRSF14 |
| **HLA COMPLEX** | B2M, HCG26, **HLA-DQA2**, RFXANK |
| **COMPLEMENT** | C1QA, C5AR1, **C6**, CFD, ITGAX |
| **IMMUNOGLOBULINS** | **IGLL5**, **IGSF10**, LILRA2, MSMB, **PIGR** |
| **INTERFERONS** | IFITM1, IFITM3, ISG20, IFI27, IFI30, TMEM91 |
| **CELLULAR IMMUNITY** | FCGR3B, FCGRT, **KLRC3**, LY6D, LY6K |
| **T- CELL SIGNALLING** | **BTN1A1**, **CD3E**, **CD3G**, KLF2, MS4A4A, **TRAT1**, TCIRG1, **VTCN1** |
| **OTHERS** | CSF3R, EMR3, FPR1, LTB4R2, MARCO, MST1P2, PTGES2, RHBG, SLPI, STAP2, SSNA1, **XKR4**, **XKR9** |

*Supplementary Table 8.* Differentially expressed genes in Parkinson’s disease versus normal skin validated by qRT-PCR

| GENE NAME | qRT-PCR log2 FC | RNAseq log2 FC |
| --- | --- | --- |
| SAA-1 | -1.7 | -2.75 |
| SAA-2 | -2.7 | -1.65 |
| HBA-2 | -1.1 | -2.05 |
| CALML-6 | -1.5 | -2.79 |
| DGCR-6L | -1.6 | -2.94 |
| CST E/M | -1.02 | -1.59 |
| OR2HR | -1.5 | -3.6 |
| ROMO-1 | -1.27 | -1.23 |
| ADAMDEC | 1.21 | 1.86 |
| HCRT | -1.08 | 2.62 |
| KLRC-3 | -1.58 | 1.99 |
| APOC-1 | 1.07 | -1.45 |

*Supplementary Table 9.* Demographic and clinical characteristic of patients with Parkinson’s Disease used in qRT-PCR analysis.

| **Variable** | **Mean ± SD or n (%)** |
| --- | --- |
| **Female gender** | 19 (51.4%) |
| **Age, years** | 69.4±7.2 |
| **Age of PD onset, years** | 61.7±8.7 |
| **Duration of PD, years** | 8.01±5.2 |
| **Clinical subtype**  Tremor-dominant  Hypokinetic-rigid dominant  PIGD | 17 (45.6%)  16 (43.2%)  4 (10.8%) |
| **MDS-UPDRS** | 62.4±25.8 |
| **HY, median** | 2.5 (range: 2-4) |
| **SE-ADL, median** | 90 (range: 55-100) |
| **MMSE** | 28.8±1.7 |
